# Late-onset bilateral lens dislocation and glaucoma associated with a novel mutation in *FBN1*

**Published:** 2008-06-30

**Authors:** Ting Deng, Bing Dong, Xiaohui Zhang, Hanjun Dai, Yang Li

**Affiliations:** Beijing Institute of Ophthalmology, Beijing Tongren Hospital, Capital University of Medical Science, Beijing, China

## Abstract

**Purpose:**

To describe the clinical and genetic findings in one Chinese family with late-onset bilateral lens dislocation and secondary glaucoma.

**Methods:**

One family including three affected members and 16 unaffected family members was examined clinically. After informed consent was obtained, genomic DNA was extracted from venous blood of all participants. Linkage analysis was performed with two microsatellite markers around the *fibrillin-1* (*FBN1*) gene (D15S992 and D15S126). Mutation screening was performed using direct DNA sequence analysis and single strand conformation polymorphism (SSCP).

**Results:**

Clinical examination and pedigree analysis revealed that four members in three generations were affected by late-onset lens dislocation and secondary glaucoma but had no signs of cardiovascular abnormality or abnormal skeletal features. By genotyping, the family showed the linkage to *FBN1* on 15q21.1. After mutation screening analysis on 65 exons of *FBN1*, a novel heterozygous missense mutation, c.2860C>T (R954C), was detected. This mutation cosegregated with the disease phenotype in the family and was not found in 100 normal controls.

**Conclusions:**

Late-onset isolated ectopia lentis with secondary glaucoma is consistent with a novel mutation in *FBN1*. Our finding expands the spectrum of *FBN1* mutations and is useful for further genetic consultation and genetic diagnosis.

## Introduction

Ectopia lentis (EL; OMIM 129600) is a dominantly inherited connective disorder characterized by lens dislocation due to stretched or disrupted zonular filaments [1]. It may also occur as a common clinical feature of several different systemic hereditary diseases such as Marfan syndrome. Marfan syndrome (MFS, OMIM 154700) is an autosomal dominant connective tissue disorder involving many systems, but its more cardinal manifestations are cardiovascular, skeletal, and ocular [1]. However, isolated EL or simple EL patients present with no cardiovascular or skeletal features of MFS [1]. By genetic analysis, both EL and MFS are linked to the same gene, *fibrillin-1* (*FBN1*) [2-4]. *FBN1* mutations have been identified in patients affected by type I fibrillinopathies, which include MFS, neonatal MFS (nMFS), MASS syndrome (mitral valve, aorta, skeleton, and skin; OMIM 604308), isolated EL, Shprintzen-Goldberg syndrome (OMIM 182212), isolated skeletal features of MFS, and ascending aortic aneurysm [2-10].

*FBN1* contains 65 exons spanning 230 kb of genomic DNA on chromosome 15q21.1. The gene encodes profibrillin-1, a 350 kDa glycoprotein. This glycoprotein is further processed to fibrillin-1, the main component of 10–12 nm extracellular microfibrils that are widely distributed in both elastic and non-elastic tissues including the skin, aorta, periosteum, cartilage, and ciliary zonules [5-8]. Fibrillin-1 contains 47 motifs with homology to the human epidermal growth factor (EGF); 43 of these also contain a consensus sequence for calcium binding (cbEGF). EGF motifs have six conserved cysteine residues that form three disulfide bonds—between C1 and C3, C2 and C4, C5 and C6. It also has seven TGFβ1-binding protein-like modules containing eight-cysteine motifs (8-Cys/TB), a two-hybrid domain, a NH_2_-terminal domain, one proline rich region, and a COOH-terminal domain [6].

Here, we reported a Chinese family associated with late-onset isolated EL and secondary glaucoma. Molecular genetic analysis of the family revealed a novel heterozygous missense mutation in *FBN1*.

## Methods

### Patients and DNA samples collection

This study was approved by the Beijing Tongren Hospital Joint Committee on Clinical Investigation. After informed consents were obtained, all participants underwent physical, ophthalmologic, and cardiovascular examinations. Ophthalmologic examinations included bilateral visual acuity, slit-lamp biomicroscopy, fundus examination with dilated pupils, and ultrasound biomicroscopy (UBM). Cardiovascular examinations included electrocardiogram and echocardiogram. Peripheral blood was obtained by venipuncture, and genomic DNA was extracted according to standard protocols.

### Linkage analysis

**Figure 1:**
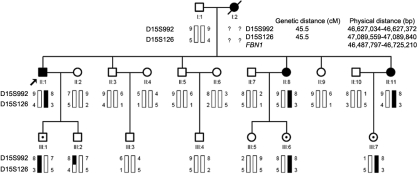
Family structure and haplotype analysis of the Chinese family with ectopia lentis. Pedigree and haplotype analysis of the Chinese families with EL showed segregation with two microsatellite markers on chromosome 15, listed in descending order from the centromeric end. Squares indicate males; circles indicate females; slashed symbols indicate that the member is deceased; solid symbols denote that the member is affected; open symbols mean the member is unaffected; open symbols with a dot in the center denote that the member is an asymptomatic carrier; arrow symbol denotes the proband.

Genotyping and linkage analysis were performed with two microsatellite markers, D15S992 and D15S126, around *FBN1*. Their detailed genetic and physical distances are shown in Figure 1. The primer sequences of D15S992 and D15S126 were obtained from the The GDB Human Genome Database. Genotyping and linkage analysis were performed as described elsewhere [11,12]. LOD scores were calculated for the two markers by two-point linkage analysis using linkage package 5.2. We modeled the disease as an autosomal dominant trait with reduced penetrance. Pedigree and haplotype were constructed using Cyrillic V. 2.0 software.

### Mutation screening of *FBN1*

The whole coding region of *FBN1* was amplified by polymerase chain reaction (PCR) from genomic DNA. Sixty-five pairs of primers for *FBN1* were used according to the articles previously published [13,14]. Nucleotide sequences were compared with the published cDNA sequence of *FBN1* (GenBank accession number NM_000138) using DNAssit version 1.0.

### Single strand conformation polymorphism

Single strand conformation polymorphism (SSCP) was used to exclude the point mutations from the normal controls. PCR amplified DNA fragments were mixed with an equal volume of formamide buffer and electrophoresed on a 12% nondenaturing polyacrylamide gel (12 ml 30% PAGE [acrylamide:bisacrylamide=29:1]; 3 ml 10X TBE; 15 ml distilled water; 600 μl 10% ammonium persulfate, 5 μl tetramethylethylenediamine). After electrophoresis, gels were silver-stained and analyzed.

## Results

### Clinical findings

We have identified a Chinese family with bilateral lens dislocation. The mode of inheritance was autosomal dominant (Figure 1). The family had 20 individuals; four of them were affected (one male and three females). As the mother of the proband passed away several years ago, we did not get her blood sample. However, from her hospital records, we inferred that she suffered the same eye disease. After clinical examinations and reviewing hospital records, we found all affected members shared almost the same clinical manifestations. All of them first experienced the sudden blurring of vision with periocular pain and congestion, then ophthalmologic examinations showed high intraocular pressure (IOP; 40–80 mmHg), corneal edema, shallow anterior chamber, and lens dislocation. All affected members underwent lens extraction, and their IOP were in the normal range after surgery. Fundus examination for three of the affected individuals showed healthy and pink optic discs with a cup/disc ratio around 0.4 (except the proband’s right eye). Physical and cardiovascular examinations presented no skeletal and cardiovascular features of MFS in any of the affected members. Their detailed clinical information is summarized in Table 1.

**Table 1:** Clinical details of three affected members and three carriers in the ectopia lentis family.

	**II:1**	**II:8**	**II:11**	**III:1**	**III:6**	**III:7**
**Age**	67	61	49	36	34	21
**Onset age**	37	54	42	-	-	-
**Ocular features**						
Best corrected Visual Acuity (R/L)	LP*/1.5	1.0/1.0	1.0/1.0	1.0/1.0	1.0/1.0	1.0/1.0
Ectopia lentis	+	+	+	-	-	-
Secondary glaucoma	+	+	+	-	-	-
Eye operation (R)	LE	ILV	ILV	-	-	-
Eye operation (L)	PI	ILV	ILV	-	-	-
Cornea	CL (R)	-	-	-	-	-
Myopia	?	+	+	+	+	+
Retina detachment	-	-	-	-	-	-
**Skeletal features**						
Height (cm)	168	160	165	174	165	162
Pectus carinatum	-	-	-	-	-	-
Pectus excavatum	-	-	-	-	-	-
Scoliosis (>20°)	-	-	-	-	-	-
Arachnodactyly	-	-	-	-	-	-
Joint hypermobility	-	-	-	-	-	-
**Cardiovascular features**						
Mitralvalve prolapse	-	-	-	-	-	-
Aortic ascendens dilatation/dissection	-	-	-	-	-	-

R represents right eye; L represents left eye; + represents positive syndrome; - represents negative syndrome; ? represents syndrome is unknown; LP represents light perception; LE represents lensectomy; PI represents peripheral iridotomy; CL represent corneal leukoma; ILS represent intraocular lens suspension; The asterisk indicates that the lens was completely dislocated into the anterior chamber, which induced corneal endothelial decompensation and corneal opacity.

### Genotyping results

This family with isolated EL was genotyped with two microsatellite markers located around *FBN1* in the 15q21.1 region. The marker results for D15S992 and D15S126 were fully informative for linkage. Haplotypes were constructed for this family to determine whether the disease was segregating with microsatellite markers. For this family, there were no affected recombinants for either of the two markers (Figure 1). Interestingly, three clinical unaffected individuals (III:1, III:6, and III:7) inherited the affected haplotype as well. As the lens luxation in this family seemed to represent a late-onset feature in the phenotype and all three of them were under 37 years old (the youngest age of onset in this family), they were obligate carriers. Their detailed clinical information was also summarized in Table 1. Therefore, the disease penetrance appeared incomplete in this pedigree. Two-point LOD scores for D15S992 and D15S126 with 60% penetrance were 0.70 (θ=0.0) and 2.36 (θ=0.0), respectively.

### Mutation analysis

**Figure 2:**
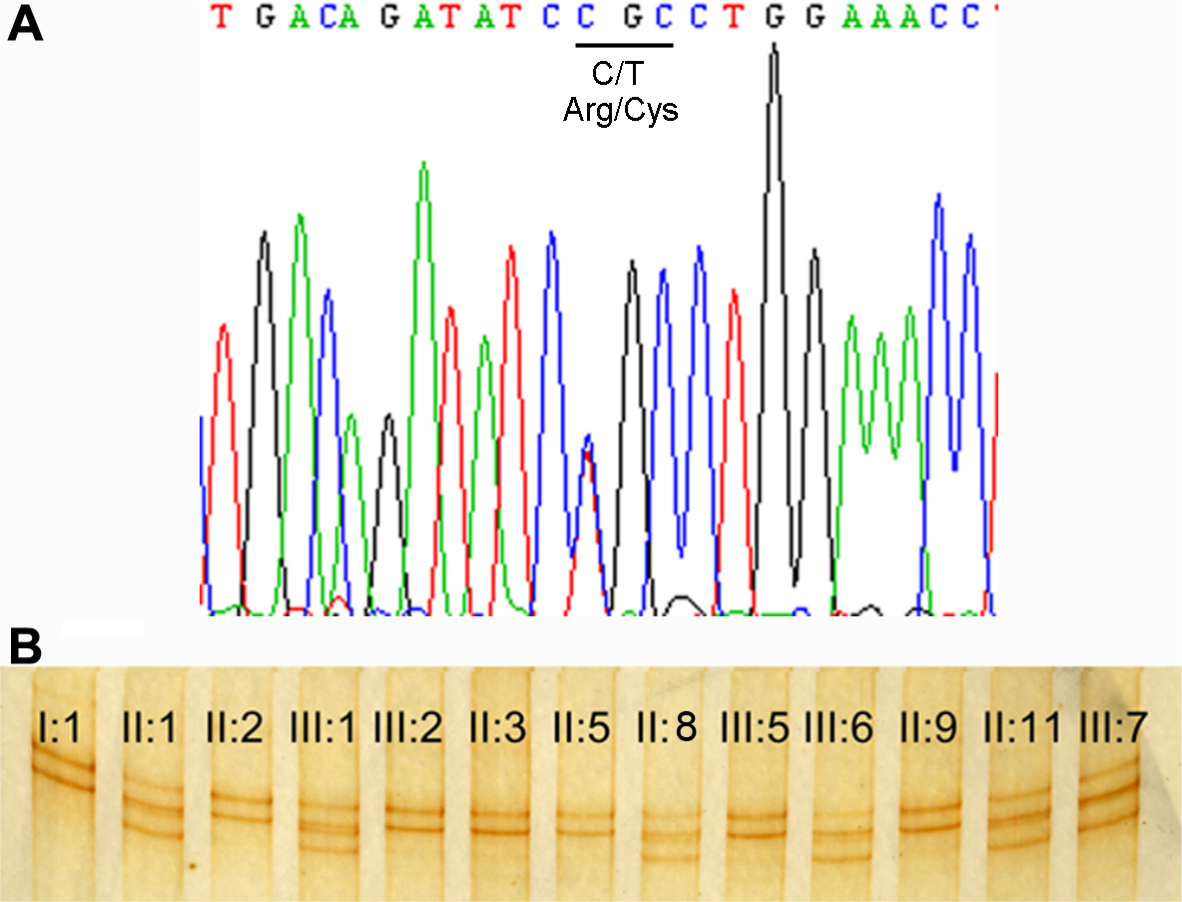
DNA sequence chromatograms and cosegregation analysis of the R954C mutation with disease phenotype. **A**: Heterozygote sequence (sense strand) shows a C→T transition in codon 954 that changed arginine (CGC) to cysteine (TGC). **B**: Single strand conformation polymorphism (SSCP) analysis shows that the mutant pattern (three bands) cosegregated with affected individuals and carriers harboring heterozygous for the C→T transition but not with unaffected individuals and spouses (two bands).

By direct sequencing the 65 exons of *FBN1*, we identified a novel base change, C>T, at position 2860 of cDNA, replacing arginine acid with cysteine at codon 954 (Figure 2A). Using SSCP analysis, this heterozygous mutation cosegregated with all affected members and affected haplotype carriers in this Chinese family (Figure 2B) but was not detected in 100 unrelated normal controls.

## Discussion

In this study, we analyzed a Chinese family with four members affected by lens dislocation along with secondary glaucoma. By genotyping, the family showed the linkage to the EL locus on 15q21.1. One novel heterozygous *FBN1* mutation, R954C, was identified in this family. The mutation, R954C, was found to cosegregate with the EL phenotype, and where three clinically unaffected members carried the mutation, they were also found to harbor the affected haplotype. This variant was not detected in 100 normal control individuals. All affected individuals showed ocular involvement only and did not meet the Ghent criteria for Marfan syndrome [6]. Diagnosis of isolated ectopia lentis was established for this family. In our review of the literature, isolated EL occurs either as a congenital disorder or as a spontaneous disorder of late onset, which is between the ages of 20 and 65 years [1,7,15]. This family’s late-onset feature (37–51 years old) is in accordance with the secondary condition. As three obligate carriers in this family were still younger than 37 years old (the youngest age of onset in this pedigree), they might develop EL in the future or present reduced penetrance or non-penetrance as reported before [7,15]. They should therefore undergo ophthalmologic surveillance at regular intervals throughout their lives.

To date, over 600 mutations in *FBN1* have been reported. However, mutations for isolated EL only take a small part (*FBN1* Universal Mutation Database [UMD]). Mutation R954C that was detected in this study is the first missense mutation in exon 24 associated with isolated EL. Mutations within the middle region (exons 24–32) of *FBN1* are usually associated with a severe form of MFS, neonatal MFS, and define a high-risk group for cardiac manifestations [5-8,16]. Mutation R954C is located in the third 8-Cys/TB modules, which contains eight cysteine residues that form four disulfide bonds—between C1 and C3, C2 and C6, C4 and C7, and C5 and C8. This mutation adds a new cysteine residue, which might destroy the disulfide bond formation and further influence the structure and function of fibrillin-1.

Early in 2002, [17] Comeglio et al. noted that mutations involving non-conserved arginine to cysteine substitution are usually associated with isolated EL. To date, nine of these types of substitutions have been identified in *FBN1* (Table 2) [13,17-29]. Seven of them including the one detected in this study definitively caused isolated EL, and the majority of them are confined to the first 15 exons of *FBN1* (5/7). Our results support the prior suggestion that non-conserved arginine to cysteine substitution is highly related to predominant EL regardless of which modules they are located [17,18].

**Table 2:** Arginine to cysteine substitutions reported in *FBN1* is related to isolated ectopia lentis.

**Mutations**	**Exons**	**Protein domains**	**IEL reported**	**Other clinical manifestations reported**	**Reference**
R62C	2	Fib-4-cys	Yes	RD, ARD, Ar, PP, En, SA	[19,20]
R122C	4	EGF-like #2	Yes	My, HS, MVP, HP, Ar, KJE, Sco, PE, TK, IH	[13,17,18,21-24]
R240C	6	Hybrid Motif No 1	Yes	JH, My, Str, HS, SA, IH	[13,17,21,23,25]
R545C	13	cbEGF-like #4	Yes	HP, JH, My, PE, PC, FC, Str, IAL, DC	[17,18,21,23,26]
R627C	15	cbEGF-like #6	Yes	HP, DBH, MVP, My, FC, DC	[18,26,27]
R954C	24	LTBP #03	Yes	Gla	this report
R1530C	37	LTBP #04	Yes	MAD, My, FC, Sc, Ar, RE, DC	[18,21]
R1832C	44	cbEGF-like #26	?	?	[28]
R2680C	63	cbEGF-like #43	No	MVP, Sco, JH, PE	[29]

Fib-4-cys: a four cysteine motif with some similarities to the Fib domain; EGF-like: EGF-like domain; cbEGF-like: calcium binding EGF-like domain; LTBP: latent transforming growth factor-β binding protein- like domain; IEL-isolated ectopia lentis; RD-retinal detachment; ARD-Aortic root dilation; Ar-Arachnodactyly; PP-pes planus; En-Enophthalmus; SA-Striae atrophicae; My-myopia; HS-hyperextensible skin; MVP-Mitral valve prolapse; HP-high palate; KJE-knee joint effusions; Sco-Scoliosis; PE-pectus excavatum; TK-thoracal kyphosis; IH-inguinal hernia; JH-joint hypermobility; Str-strabismus; PC-Pectus carinatum; Gla-glaucoma; MAD-mild aortic dilation; FC - flat cornea; IAL-increased axial length; RE=reduced extension at the elbows (<170°); DC=dental crowding; DBH-disproportionate body habitus.

Another clinical feature of this family is glaucoma, which is relatively common and a serious complication of ectopia lentis [1]. According to the literature, glaucoma usually occurs more frequently in spontaneous late subluxation of the lens than in the congenital type [1]. We concluded that the pathogenesis of glaucoma in this family was due to lens-induced pupillary block.

In summary, we described a novel non-conserved arginine to cysteine substitution in exon 24 of *FBN1* that is associated with late-onset isolated EL and secondary glaucoma. Our results further expanded the mutation spectrum of *FBN1* and provided useful genetic consultation and genetic diagnosis for this family.
